# Medical errors: Healthcare professionals’ perspective at a tertiary hospital in Kuwait

**DOI:** 10.1371/journal.pone.0217023

**Published:** 2019-05-22

**Authors:** Zamzam Ahmed, Mohammad Saada, Alan M. Jones, Abdullah M. Al-Hamid

**Affiliations:** 1 School of Pharmacy, University of Hertfordshire, Hatfield, United Kingdom; 2 School of Pharmacy, University of Birmingham, Edgbaston, United Kingdom; Nord University, NORWAY

## Abstract

Medical errors are of economic importance and can contribute to serious adverse events for patients. Medical errors refer to preventable events resulting from healthcare interactions, whether these events harm the patient or not. In Kuwait, there is a paucity literature detailing the causes, forms, and risks of medical errors in their state-funded healthcare facilities. This study aimed to explore medical errors, their causes and preventive strategies in a Kuwait tertiary hospital based on the perceptions and experience of a cross-section of healthcare professionals using a questionnaire with 27 open (n = 10) and closed (n = 17) questions. The recruited healthcare professionals in this study included pharmacists, nurses, physicians, dentists, radiographers, hospital administrators, surgeons, nutritionists, and physiotherapists. The collected data were analysed quantitatively using descriptive statistics. A total of 203 participants filled and completed the survey questionnaire. The frequency of medical errors in Kuwait was found to be high at 60.3% ranging from incidences of prolonged hospital stays (32.9%), adverse events and life-threatening complications (32.3%), and fatalities (20.9%). The common medical errors result from incomplete instructions, incorrect dosage, and incorrect route of administration, diagnosis errors, and labelling errors. The perceived causes of these medical errors include high workload, lack of support systems, stress, medical negligence, inadequate training, miscommunication, poor collaboration, and non-adherence to safety guidelines among the healthcare professionals.

## Introduction

Medical errors (MEs) are one of the common causes of iatrogenic adverse outcomes in the healthcare industry. A ME is defined as a failure to achieve planned actions (errors of execution) or using wrong plans to attain an objective (errors that result due to planning) [1]. An unintentional act (either of commission or omission) or an act that fails to achieve its planned outcome is another definition for MEs [2]. It is argued that often, there are circumstances beyond the control of the healthcare provider that influence patient outcomes [3]. For instance, a patient may present with an unknown allergic reaction after receiving a new medication. In this case, the allergic reaction is the unexpected or unplanned outcome, yet it cannot be holistically argued that the outcome is attributable to ME.

Patient safety is a basic patient right and should be ensured during hospital visits or admission [4]. Furthermore, it is the duty of healthcare professionals (HCPs) and institutions to ensure patient safety, improve treatment outcomes and reduce adverse events (AEs) [4]. A failure to provide safety may result in death, disabilities, poor health outcomes, increased costs and legal issues [4]. It is noted that MEs and AEs are inevitable in almost all healthcare settings [5]. A recent report on healthcare quality advocated the need for a thorough approach to MEs in the Middle East [6]. In addition, the authors emphasised the need for learning and identifying MEs through voluntary and mandatory reporting systems [6]. Undertaking such an approach would be essential in addressing significant AEs that occur in day to day activities in the healthcare sector.

In the light of the above study characteristics and demographic trends in the Middle East regarding MEs, it is important to explore the potential causes and preventive measures of MEs. Exploring MEs from the perspective of the HCPs is crucial in early mitigation of potential errors. Preventing MEs will be of economic importance to the healthcare industry in terms of reducing unnecessary rehospitalisations, and diagnosis [7]. Moreover, MEs prevention is important in promoting patient safety culture (PSC) and eliminating financial burdens on healthcare institutions, and families of the affected patients [4, 8]. In addition, prevention of MEs can help mitigate other adverse outcomes such as permanent disability, complications, and death [9, 10].

There is a paucity of data available on MEs in Kuwait’s healthcare industry. There have been very few academic studies in this field in Kuwait [10, 11]. A study conducted in 2014 evaluated PSC in Kuwait and reported that participants (nurses, physicians and administrative staff) rated patient safety at their workplaces highly, with 74.1% reporting no events that compromised patient safety in the last one year [10]. Only 13.0% reported one or two AEs within the same time period.

Ali and colleagues found that when assessing PSC in Kuwait, the hospital management lacked critical unit-level systems such as non-punitive responses, open communication channels, and staffing important to improving patient safety [11]. Similarly, the study conducted by Ghobashi and colleagues revealed in their research that respondents identified non-punitive response to errors, communication openness and adequate staffing was essential in ensuring patient safety [10]. However, the respondents indicated that they did not compromise patient safety to get more work done implying that the perception of patient safety among medical workers was high. While another study explored PSC in Kuwait among hospital staff [11], Ghobashi and colleagues only investigated awareness among primary healthcare providers about PSC [10].

The present study aims to ascertain the perceptions of HCPs about the causes and preventative measures of MEs in a Kuwait tertiary hospital.

## Materials and methods

### Research strategies and design

In this cross-sectional study, a quantitative research approach was used including open-ended (n = 10) and closed (n = 17) survey questions. The use of quantitative surveys was preferred for this study because it was a versatile design, allowing for a variety of methods to recruit participants and collect data using various tools and instruments.

### Research setting and participant sampling technique

The research setting was limited to a Kuwaiti tertiary hospital where the research participants included HCPs from all the departments. A random sampling technique was employed to recruit participants from each department for inclusion in the study. Random sampling ensured that everyone in the target population had an equal opportunity of being drawn into the research. By using random sampling the likelihood of bias during the selection of participants was minimised and sampling errors were reduced [12]. A sample of 203 participants (due to resources, time, and study objectives) comprising of HCPs from various departments such as pharmacy, nursing, physicians, and administrators were recruited for the study through random sampling.

### Data collection tool

The tool for data collection was a self-administered open and closed-ended questionnaire (S1 Appendix). The questions were written by MS, reviewed by the research team then translated into Arabic and further refined in the pilot stage described below. The questions were grouped under three sections, each exploring a specific theme.

Section one of the questionnaire sought to collect demographic profile of the respondents, including the participants’ age, gender, nationality, qualifications, position, the department they practised in and years of experience.

The second section of the questionnaire assessed the knowledge of the participants regarding MEs and inquired whether they had witnessed or had been part of a ME and the consequences. Finally, the last section of the questionnaire explored the attitudes and opinions of participants about initiatives to minimise or prevent MEs. The different parts of the questionnaire are summarised in the supplementary material.

### Data collection procedure

The questionnaire was self-administered, and participants were required to take the survey either online (using SurveyMonkey) or on paper format. The distribution of questionnaires was done online and in such a way that the researcher was not in a position to tell who completed the survey questionnaire. The link to the survey was emailed to participants. No identifiable personal data was collected during the surveying process. The questionnaire was also printed and made available at reception desks from where respondents could collect them and also return after completion.

### Data analysis

Descriptive statistics were used to summarise aspects of the data to provide information about the sample as well as the population from which it was drawn [12]. Frequencies and percentages were used to summarise the data. Frequencies are commonly used with discrete variables. Relative frequencies were used to show the proportions of the sample and consequently, the population, in terms of age, gender, length of work, and area of specialisation. Moreover, frequencies and percentages were also used to analyse the data from the scale-based questions where respondents selected one answer from given options. The number of respondents who gave a certain response out of the total number of respondents were provided to show the perspectives of the healthcare professionals towards a certain metric. The summaries derived from the descriptive analysis were presented in charts and tables.

### Questionnaire pilot study

A pilot study was conducted with ten respondents due to the study resources. The pilot study aimed to test the face and content validity of the questionnaire. The pilot study also assessed the research protocols and recruitment strategies [13]. The pilot survey also enabled the researcher to make any modification needed and clarify vague questions. A total of five questions were modified as a result of the pilot study.

### Potential research risks

The most significant risks associated with this research are linked to the aspects of confidentiality. Past literature studies have shown that there are feelings of shame, guilt and panic after the occurrence of MEs among HCPs [14]. HCPs may be afraid for their reputation, career, future, and even their medical licenses if they admit to committing MEs [15]. Therefore, there might be a risk of respondents refusing to participate due to guilt and fear as well as uncertainty about confidentiality. The researcher informed the participants that their confidentiality would be guaranteed, data obtained anonymously.

### Potential ethical concerns

Ethics Committee approval was obtained from the Kuwait Ministry of Health and the University of Hertfordshire, UK prior to commencement of the study. Participants were informed that taking part in the study was voluntary and that they were free to withdraw from the study at any time. No identifiable or personal data was collected, and confidentiality was guaranteed as discussed above. The participants were assured that the data was collected for academic research only and that the collected information would be securely stored in a password encrypted computer to prevent unauthorised access in efforts of ensuring information confidentiality.

## Results

### Demographic characteristics

A total of 203 out of the 206 participants approached responded representing a response rate of 98.5%. Out of the 203 respondents, there were a total of 84 (41.4%) male participants and 119 (58.6%) female participants (Table 1).

**Table 1:** Demographics and characteristics of the healthcare professionals (HPCs) included in the study (n = 203).

Characteristic	Variables	n (%)
Gender	Male	84 (41.4)
	Female	119 (58.6)
Age (years)	<25	13 (6.4)
	25–29	65 (32)
	30–39	84 (41.4)
	40–49	30 (14.8)
	50–59	7 (3.4)
	>59 NR	4 (2) 124 (61.1)
Educational level	Doctorate	15 (7.4)
	Masters	36 (17.7)
	Bachelors	115 (56.7)
	Diploma	37 (18.2)
	Certificate	0 (0)
Specialism*	Radiographer	11 (5.4)
	Administrator	8 (3.9)
	Dentist	8 (3.9)
	Pharmacist	102 (51.7)
	Surgeon	2 (1.0)
	Nutritionist	17 (8.3)
	Physiotherapist	4 (2.0)
	Nurse	4 (2.0)
	physician	16 (7.8)
	Others e.g. laboratory technicians, oncologists NR	28 (13.7) 5 (2.5)
Experience (years)	<1 yr	41 (20.2)
	1–3 yrs	15 (7.4)
	3–5 yrs	50 (24.6)
	5–10 yrs	40 (18.2)
	>10 yrs	57 (28.1)

NR: not reported,

*: Specialism: area of participant’s expertise

As further shown from Table 1, the majority of the participants (41.4%, n = 84) were aged between 30 and 39 years followed by those that fell in the age bracket of 25–29 years, 40–49 years, under 25 years, 50–59 years, and above 59 years respectively. In terms of career occupation, most participants that took part in the survey were pharmacists (51.7%, n = 102), followed by nutritionists (8.3%, n = 17), physicians (7.8%, n = 16), radiographers (5.4%, n = 11), administrators (3.9%, n = 8), and dentists (3.9%, n = 8). Participants were also asked to share their opinion in terms of job satisfaction at their present workplace. The feedback revealed that 64% (n = 130) of the participants were satisfied, whilst 36% (n = 73) were not satisfied with their work, respectively.

### Common medical errors in Kuwait’s tertiary hospital

A total of 44.6% of respondents confirmed that they had encountered potential MEs while 55.4% had not experienced any MEs in their practice. Table 2 shows the potential MEs that are commonly encountered in Kuwait healthcare facilities according to the participants. As shown in Table 2, the main common types of MEs include making wrong dispensations, prescriptions, dosage, explanation/descriptions, diagnosis, and drug formulation. According to the participants, other MEs such as dispensing wrong medical results from lack of enough time to review orders for appropriateness which results in increased likelihood among care providers to make mistakes. Also, lack of tools to help clinicians to check drug-drug interactions especially in polypharmacy prescriptions resulted in high MEs.

**Table 2:** Main medical error themes identified by healthcare professionals (HCPs).

Medical errors (MEs)	Response (n, %)
Dispensed medication with incomplete instructions	19 (33.5)
Prescribed drugs to the incorrect patient / no check for patient drug allergies	19 (33.5)
Incorrect dose or overdose for adults and paediatric patients	18 (31.8)
Wrong administration of medicines to patients	16 (30.0)
Wrong explanation of medication usage	15 (29.5)
Wrong diagnosis when first admitting the patient	15 (29.5)
Similar medication brands–difficult to distinguish	14 (27.1)
Drug formulation unsuitable for patient condition	11 (24.4)
Dispensing antibiotics very often without appropriate tests conducted	9 (21.7)
Potential errors when entering patient data	8 (20.6)

The participants were asked about relationship between colleagues as they may have a bearing in mitigating potential MEs. The workplace relations among the participants ranged from perfect (26.6%), satisfactory (41.8%), compromised (29.8%), to bad (1.3%). It was evident from 61.4% of the participants that the state of the relationship with other colleagues largely affects the credibility of the service that individuals provide, and thus the potential for MEs. However, 38.6% of the participants expressed that the state of workplace relationships does not affect or compromise service delivery.

### Where medical errors occur

The questionnaire aimed to identify areas where MEs commonly occurred in the medical facility in Kuwait. As indicated in Table 3, the participants reported that the common areas where MEs occurred include the emergency room (57.0%, n = 112), medical wards (43.3%, n = 86), operation rooms (33.1%, n = 66), Intensive Care Units (ICUs) (17.8%, n = 35), and while other locations (17.8%, n = 35) account for the remaining MEs. In addition, Table 3 also shows the additional areas where the MEs were likely to be reported including the out-patient department, clinics, during hospitalisation, dietary department, negligence by nurses who do not take care of the patient, pharmacy, and during diagnosis.

**Table 3:** The main areas where medical errors are commonly encountered.

Hospital Department	n (%)	*Others (in detail)	n (%)
Operating room	66 (33.1)	OPD (out-patient department)	8 (4.0)
Emergency room	112 (57.0)	Clinics	7 (3.5)
Wards	86(43.3)	During hospitalisation	6 (3.0)
ICU	35 (17.8)	Dietary department	4 (2.0)
Others	35 (17.8)	Infection control is not sufficiently effective	4 (2.0)
		Nurses do not take care of patient	4 (2.0)
		Pharmacy	4 (2.0)
		diagnosis	3 (1.5)
		Anywhere in the clinic	3 (1.5)

* Open ended responses

Participants were asked to share their views on who holds the largest responsibility for the regular MEs encountered in the hospital environments. The respondents noted that fellow colleagues (49.7%) were to be held accountable for MEs, followed by the system used to run the hospital facility (40.3%), and the hospital administration (27.0%). In addition, other participants (20.8%) also expressed that patients were to blame for MEs, while other respondents indicated that various departments were responsible for MEs (7.6%).

### Causes of medical errors

The survey sought to identify the most common causes of MEs. Table 4 shows the main responses on the causes of MEs in Kuwait based on participant insights.

**Table 4:** The common causes of medical errors in Kuwait.

Main Cause of MEs	n (%)	*Other causes of MEs	n (%)
Miscommunication between patients & HCPs	124 (62.7)	High workload	23 (11.6)
Miscommunication between HCPs	70 (35.4)	Stress & long duty hours	19 (9.5)
Lack of rest breaks for HCPs	59 (30.0)	Lack of electronic systems	19 (9.5)
Others	30 (15.3)	Diagnosis / efficiency of doctors	17 (8.5)
		Lack of attention / carelessness	17 (8.5)
		Untrained personnel	16 (8.0)
		Ignoring / Negligence	16 (8.0)
		Lack of national prescribing guidelines	8 (4.0)
		Lack of experienced administrative workers	7 (3.5)

* Open ended responses

### Potential impacts of medical errors

We aimed to identify the participants’ views about the potential negative impact that they had encountered in the healthcare centre as a result of MEs. Table 5 summarises the potential impacts of MEs from the participants’ perspectives.

**Table 5:** The potential impact of medical errors.

ME impact	n (%)
Death of patient	41 (20.8)
Side effect to the patient	64 (32.1)
Hospital (re)-admission treatment	66 (33.3)
No negative effects	70 (35.2)
Other	5 (2.5)

Participants were asked to estimate the frequency of the MEs they have encountered and the nature of their occurrence at their workplaces. The majority of participants (60.5%) indicated that they had encountered MEs on rare occasions compared to 15.3% of the participants that had experienced MEs often, 11.5% who had not encounter errors, and 12.7% of the participants that had never encountered MEs.

### Potential solutions to medical errors

We asked participants to identify possible mitigation strategies that could be used to address the potential MEs identified. A range of different suggestions were provided including reporting through incident reports (68.7%), taking advice from colleagues that were more experienced (27.3%), and ignoring the incident (2.7%). Other strategies (1.3%) such as reaching out to the patient before taking the medication and engaging with colleagues to improve service delivery were used to reduce potential MEs.

When participants asked if they were personally involved in MEs, only 5.3% of participants reported that they had committed a mistake that led to disability or death of a patient while the majority of participants (94.7%) had not. Participants were asked about their views on the potential role that health institutions have in the reduction of MEs. The participant responses are summarised below:

1. Participants noted that health institutions could facilitate the creation of awareness (57.3%), through seminars and workshops for healthcare providers.
2. In addition, 50.7% of the participants noted that encouraging workers and auditors to report MEs was also an important avenue that healthcare institutions can use to reduce MEs.
3. A total of 64.7% of the participants felt that health institutions should be more proactive in terms of performing regular analysis and evaluations. Such an approach would help deal with MEs through the analysis to find out the cause of the problem and establish a system for not repeating this error.
4. A total of 44.7% of the participants express that the problem of MEs can be mitigated if health institutions created a better working environment for workers to reduce working hours and reconsider the system of shifts and considering to reduce the number of patients who admitted to the hospital.
5. Other potential approaches (as expressed by 5.3% of the participants) that can be used by health institutions to mitigate against MEs include (i) encouraging communication between departments as well as and making people accept the fact that discussing errors to correct them will benefit the overall outcome for patients. (ii) All workers in the health field must be held accountable and submitted for investigation and legal accountability without exception. (iii) encourage reporting by the workers and other stakeholders, and (iv) reducing workload or increasing the number of HCPs to reduce workload and give employee flexible work schedules that help them achieve work-life balance.

Potential initiatives by health institutions that may aid a reduction in ME incidents included the following as noted by the participants; raising awareness about medical responsibility; encouraging employees and auditors to report; perform regular analysis and evaluation; creation of a conducive working environment. The following initiatives were also noted by fewer participants; encourage communication between all departments, emphasising that every healthcare worker should be responsible, encourage reporting and discussing possible errors, creation of further hospitals to reduce patient populations per hospital.

Participants were asked if experience and training through workshops and other learning models can help the care providers improve their accuracy when serving the patients. Majority of the participants (94.6%) noted that training and experience were important compared to 5.4% who felt that training did not influence the HCPs’ service delivery to the patients.

### Barriers to reporting

Nearly half of the survey participants (45.3%) indicated that they report errors if and when they occur, while the remaining 54.7% of the participants noted that they do not report errors. The findings of the survey showed that reporting of MEs may be hindered by various factors. The participants reported some of the hurdles they encounter when reporting MEs can be attributed to organisational culture (56.5%), lack of knowledge (47.6%), and complex incidence reporting forms (38.1%).

Moreover, the additional hurdles that participants identified as alternative hindrances to reporting of MEs include: The fear ME reports will be used to blame other departments, Lack of knowledge about the need and importance to write incident reports, People feel discouraged when they report an error, and they do not see an end result. Fear of legal liability and prosecutions, staff are afraid of legal action, Fear of the consequence that may result from ME, Lack of seriousness in dealing with medical accidents, Some of the staff ignored MEs and indifference, Lack of feedback and fear of consequences.

Participants were asked to suggest strategies that can be embraced to reduce the effect of the MEs on the patients’ health. Some of the recommendations included:

1. The need to disclose the information about the error to the patients as suggested by 72.3% of the participants.
2. According to 50.7% of the participants, the management should give the patient’s means of assessing the effectiveness of assistance following MEs.
3. The other 4.7% of the participants advocated that the reduction of MEs can be achieved through:
  - Double checking records before making final decisions.
  - Having an end result to the reporting and have a response from the administration and the directors which may show that they care to change things into a better environment.
  - Promoting a culture of transparency, dialogue, and openness.
  - Rejecting temptations of covering up or favouring colleagues that have made mistakes, reducing workload in the hospital.
  - Hiring HCPs with sufficient knowledge and experience in their fields.

## Discussion

MEs play a significant role in influencing the safety of patients in Kuwait. The research focus was to investigate the triggers of MEs and strategies that can be adopted and implemented to reduce future occurrences of MEs.

In line with research Objective 1, the data drawn from the present research revealed that the frequency of MEs in Kuwait is high. 60.5% of the survey participants indicated that they have encountered MEs on frequent occasions and an additional 15.5% of the participants that experience MEs often. In total, 76.0% of the participants have experienced MEs on a regular basis. However, only few participants reported personal involvement in MEs. This could be explained by the guilt and fear associated with MEs.

The common types of errors identified were prescribing errors, nursing errors, pharmacist errors, and laboratory or diagnostic errors. A study conducted in 2009 found similar trends in a state hospital in Ghana where prescribing errors (e.g. incomplete prescription, drug-drug interactions and incorrect medical) and nursing errors (i.e. wrong route of administration, wrong dose, and omission of medical) largely contributed to 74% of all MEs in public hospitals [16]. Similarly, another study found that MEs such as dosage, wrong descriptions, and dispensation accounted for 47.0% of MEs in the UK [17].

The present study findings are in agreement with the previous literature findings on the high frequency of MEs in the healthcare settings in both developed and undeveloped countries. For example, the HCPs confirmed that they often encountered MEs in almost every department both in the outpatient and inpatient facilities. The top five MEs reported from this study on Kuwait tertiary hospital included (i) dispensing medical with incomplete instructions to patients (33.5%); (ii) prescribe drugs to the wrong patients (33.5%); (iii) giving wrong dosage (31.8%); (iv) wrong route of administration (30.0%); and (v) misdiagnosis (29.5%). Combined, these five MEs accounted for 60.3% of all the MEs that were identified by the participants in Kuwait. Compared to published literature, the frequency of MEs appears higher than that reported in the UK and slightly lower than the values reported by Ghanaian public hospitals. Moreover, this frequency seems higher than the global average of 33.5% [18] and as well as above the 18.0% frequency reported in the United States or the 27.0% reported in the European Union [19].

As observed from the collected data, MEs led to different AEs with 20.9% of the errors resulting in the death of the affected patients. Furthermore, MEs were also associated with 32.3% of life-threatening conditions and side-effects, while 32.9% of the MEs contributed to prolonged hospital stays and re-admissions. Therefore, it is evident that the frequency of MEs is relatively high in Kuwait similar to reports from past literature findings.

For example, in the European Union, it was estimated that hospital readmissions and hospitalisations as a result of MEs account for between 8% and 12% of all reported cases [19]. In addition, a study found that 21% of US citizens are readmitted as a result of MEs resulting from AEs. Similarly, Ali and colleagues reported that between 11% and 25% of the patients in the Middle East experience AEs due to wrong prescriptions, misdiagnosis, or medical dispensation [11]. In conclusion, it can be noted that the frequency of MEs and subsequent AEs in Kuwait is currently higher compared to the frequency reported in developed countries.

The study also sought to determine the potential triggers and risk factors for MEs in Kuwait. The participants confirmed that there are several causes of MEs in healthcare settings which are as a result of miscommunication between medical providers and the patients, poor communication among the staff members such as between the doctors and the pharmacists, workplace fatigue, and carelessness and lack of attention [20].

The current study findings are in line with the past literature on the main factors that contribute to MEs in hospital environments. For example, a study reported that one of the common risk factors for MEs is inadequate knowledge and training on prescribing skills for care providers. In the process, potential errors such as over or under dosage can result when prescribing medicals to patients [21]. Another study also reported that lack of in-depth experience and knowledge about pharmacological interventions among nurses and physicians can be a potential risk factor for MEs [11]. A study conducted in 2014 found that lack of compliance among patients regarding administration and prescription guidelines further increased the risk of MEs [5]. The literature findings are also in support of the present research results as a growing body of knowledge confirm the impact that lack of reporting on AEs, heavy workload, and miscommunication among care providers has on increasing the risk of MEs [12, 22].

Moreover, the findings from this research further echo the literature findings in that the causes of MEs are largely attributed to two factors—human and organisational aspects. The human factors include ignorance, lack of experience, training, carelessness, workload, and lack of attention. In contrast, organisational factors include limited staffing levels, work schedule, workload, lack of administrators, and lack of systems such as information technology that can help streamline and improve communication between different hospital departments.

### Identification of the severity of medical errors among patients

The participants reported that on most occasions, MEs had contributed to negative impacts including increased cost of care and prolonged hospitalisation. Moreover, there was a large sector of the participants who indicated that MEs have often led to life-threatening side effects and overall poor service delivery. In the worst-case scenario, MEs have contributed to the death of patients accounting for 20.9% of the cases. As such, the severity of MEs ranged from often (60.5%), rare (15.3%), less often (11.5%) to never (12.7%).

The literature has widely documented the severity of MEs with a study indicating that such errors largely occur during drug administration and formulation processes [9]. The study participants confirmed their experience with MEs and noted that common inconsistencies develop during communication, authorisation, and prescribing due to labelling errors and dosage formulation. The situation largely occurs in outpatient clinics than in the inpatient clinics [23]. However, the workload can also increase the severity of MEs where the level of concentration among care providers may be affected by high patient number, fatigue, and individual stress.

A study by Grasso et al. identified the clinical consequences of severe MEs and noted challenges affected patients become exposed to in terms of cost of care, prolonged hospital stays, and complications [24]. Other researchers have documented increased mortality attributed to high severity MEs [25]. The situation is worsened by poor coordination and lack of training programs to educate HCPs on the importance of reporting MEs once they occur [26]. In addition, most healthcare providers lack a rating system that can be used to identify MEs and other AEs [27, 28].

Strategies that can be used to mitigate and prevent potential cases of MEs in Kuwait were also identified. Some of the initiatives that can be adapted to reduce MEs include encouraging employees to embrace incident reporting, consulting with more qualified and experienced colleagues during uncertain procedures, educating patients on the use and effect of different medications, and collaborating with colleagues to improve service delivery. The participants identified various approaches that can be used by the healthcare institutions to prevent potential MEs in future service delivery.

Tang and colleagues advocate for the need to have in place pro-active management processes aimed at reducing MEs in healthcare facilities [29]. A pro-active risk management system like the use of root-cause analysis can help reduce and prevent potential AEs. The root-cause analysis entails an analytical system that can be used to identify underlying risks that facilitate care providers, patients, and other stakeholders to commit avoidable errors [29]. Stubbs and colleagues also advocate the use of technology systems to increase patient safety through computerisation where errors of prescription, medications, synchronising data between departments, and information technology can be used to improve communication and reduce potential bias resulting from mislabelling, wrong spellings, and poor handwriting [30]. Other literature suggests that automation may play a role in improving safety, reducing errors, and avoiding human limitations when delivering care [31].

However, the use of automation and other system improvement strategies aimed at reducing potential MEs will depend on addressing a number of hurdles first. Some of the potential hurdles that should be addressed before implementing the different strategies to reduce MEs include: Encouraging anonymous reporting to eradicate the potential fears among healthcare providers that incident reporting can be used to blame other departments; Educating HCPs on the need and importance to write the incident reports; Initiating policies to act on and implement past findings on MES so as to encouraging care providers to continue reporting errors when they occur; Embracing serious guidelines when dealing with medical accidents; Providing feedback on progress made in dealing with MEs.

However, the current study has some limitations. First, the study was conducted in one hospital and pharmacists made up the majority of the sampled respondents therefore, the views expressed may not be generalisable. Nonetheless, the pharmacists comprise the majority of HCPs in addressing the shortage of care providers in most countries; both in the public and private medical facilities hence there were more pharmacists among respondents. Despite having fewer representation from nurses and physicians, a substantial input from a wide range of HCPs was obtained which gives more comprehensive insights and opinions on MEs in Kuwait tertiary hospitals. Second, the research largely focused on opinion and views shared by HCPs therefore, future studies should include the views and insights of patients and their families. Third, using qualitative methods such as interviews may have provided more in-depth responses. Nevertheless, the current study design allowed accessing a large sample of respondents and suited the present study objectives. Finally, we have designed the study tool which was piloted and assessed on face and content validity. Further assessment of the tool construct validity and reliability is required in the future.

## Conclusions

Across the global healthcare sector, MEs have been attributed to AEs, increased costs, and overall poor care delivery. This research identified the main perceived causes of MEs and the strategies that can be adopted to mitigate the identified challenges. In line with the insights drawn from the HCPs in Kuwait, it was observed that the frequency of MEs in a Kuwait tertiary hospital is high. Some of the reasons perceived to contribute to the high MEs include the inadequate experience of prescribing, limited pharmacological knowledge among some care providers, and miscommunication among care providers and with their patients. The growing random cases of MEs largely contributed to prolonged hospital stays, AEs, life-threatening complications, and even death.

Some of the measures that can be used by the hospital management and care providers to address the growing ME events include increasing awareness about MEs and the need for incident reporting, introducing training and learning workshops, and improving ME reporting. Other effective strategies that can be used to prevent MEs include undertaking a regular assessment of MEs and their impact on care delivery, promoting training programmes and intensive quality assurance measures for all HCPs. In conclusion, the findings of this study are in line with the postulated hypothesis in that healthcare professionals’ perspectives on MEs is crucial in identifying important insights about MEs and how the identified errors can be addressed.

## Supporting information

S1 Appendix (DOCX)
