# Supplementary figures and images for: Medical errors: Healthcare professionals’ perspective at a tertiary hospital in Kuwait
Source: PLoS One. 2019 May 22;14(5):e0217023. doi: 10.1371/journal.pone.0217023 (PMC6530889; doi:10.1371/journal.pone.0217023)

**Supplementary material:**


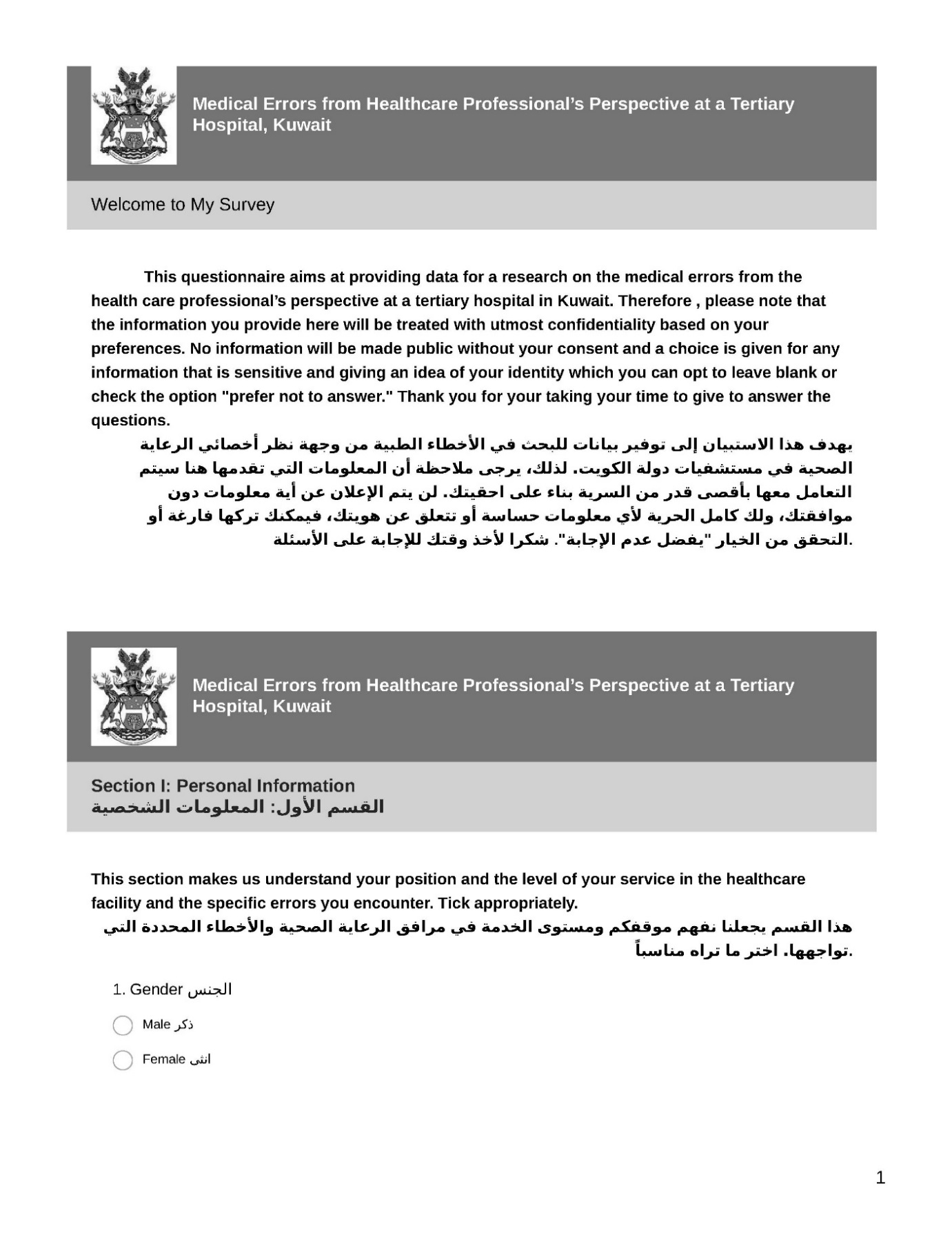


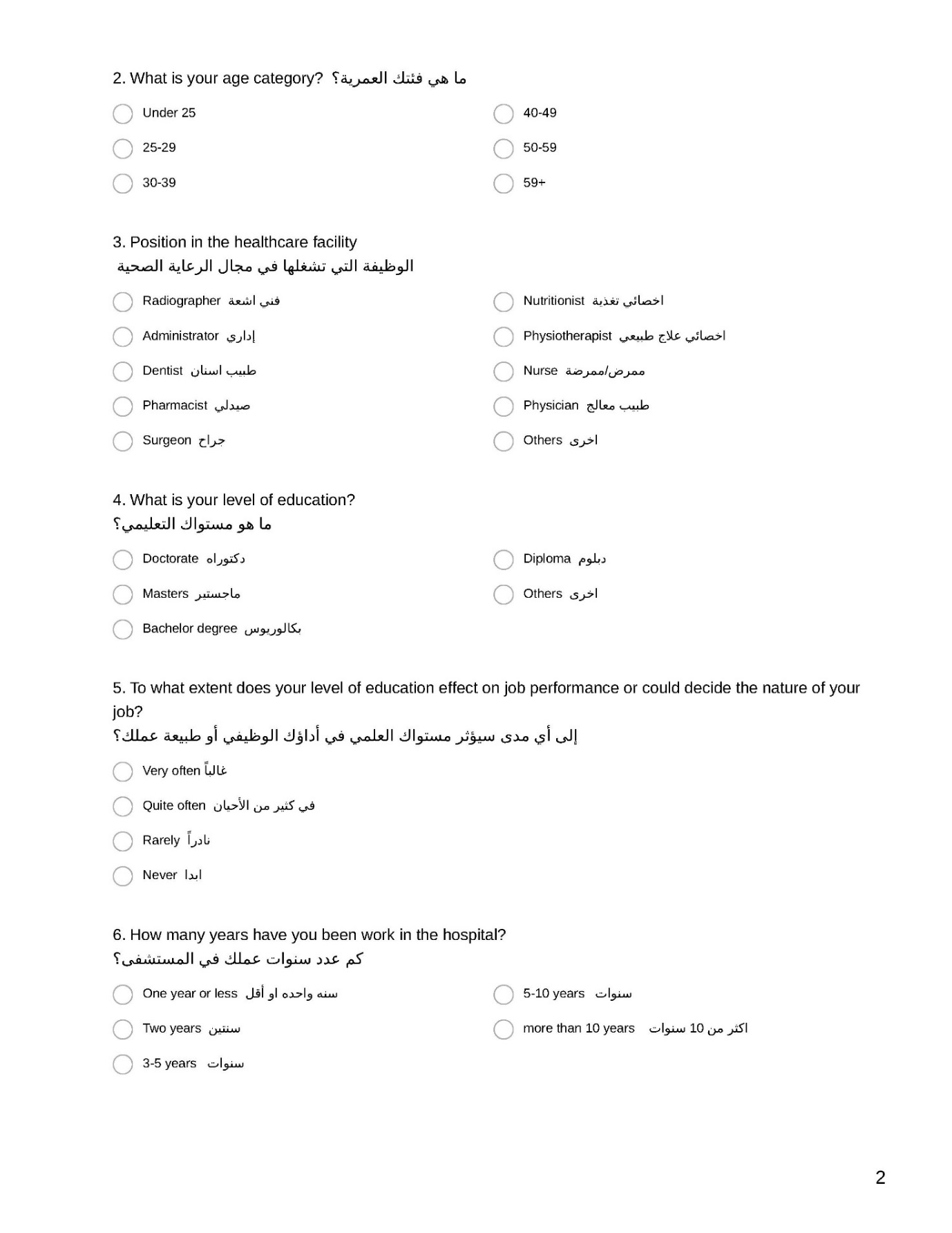


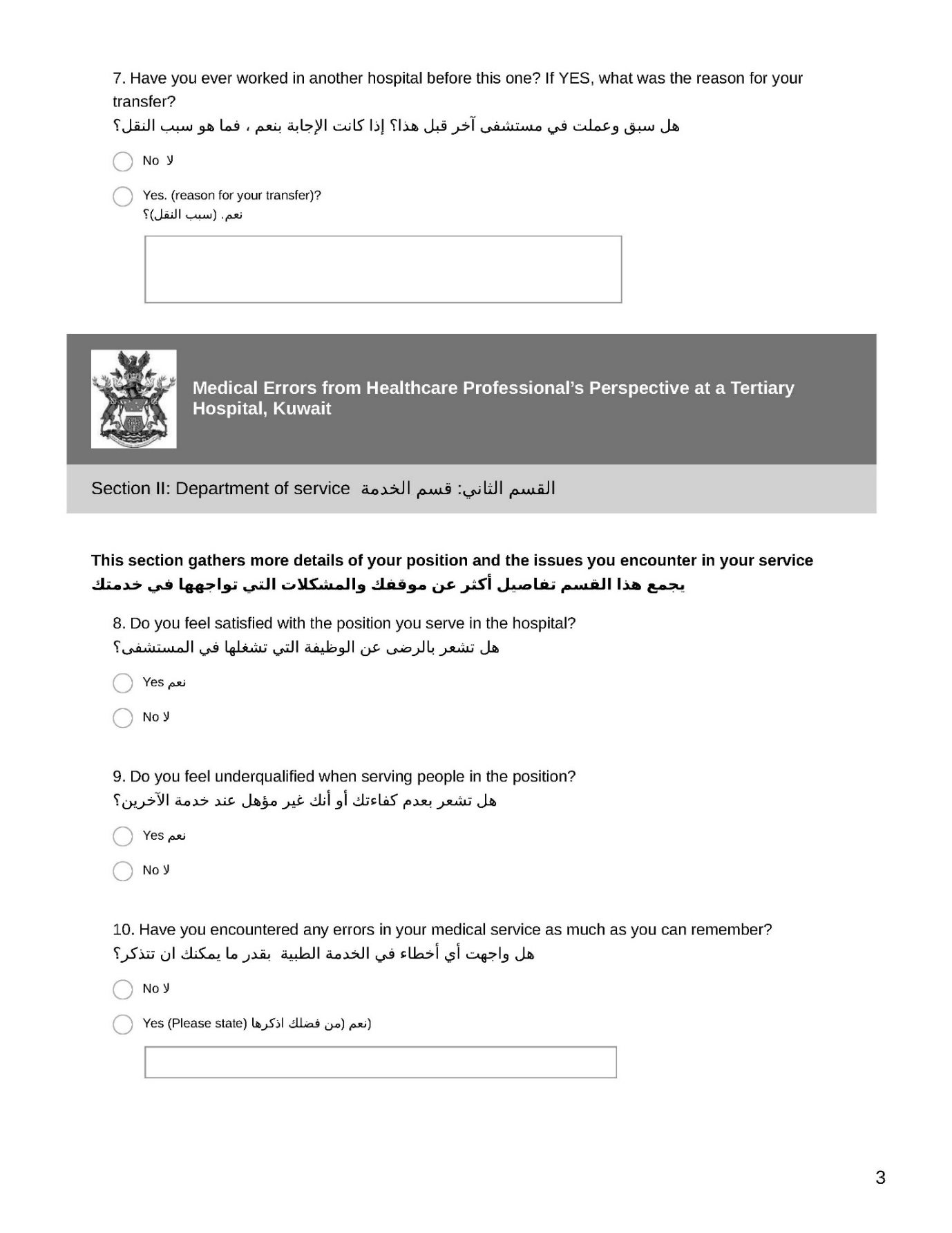


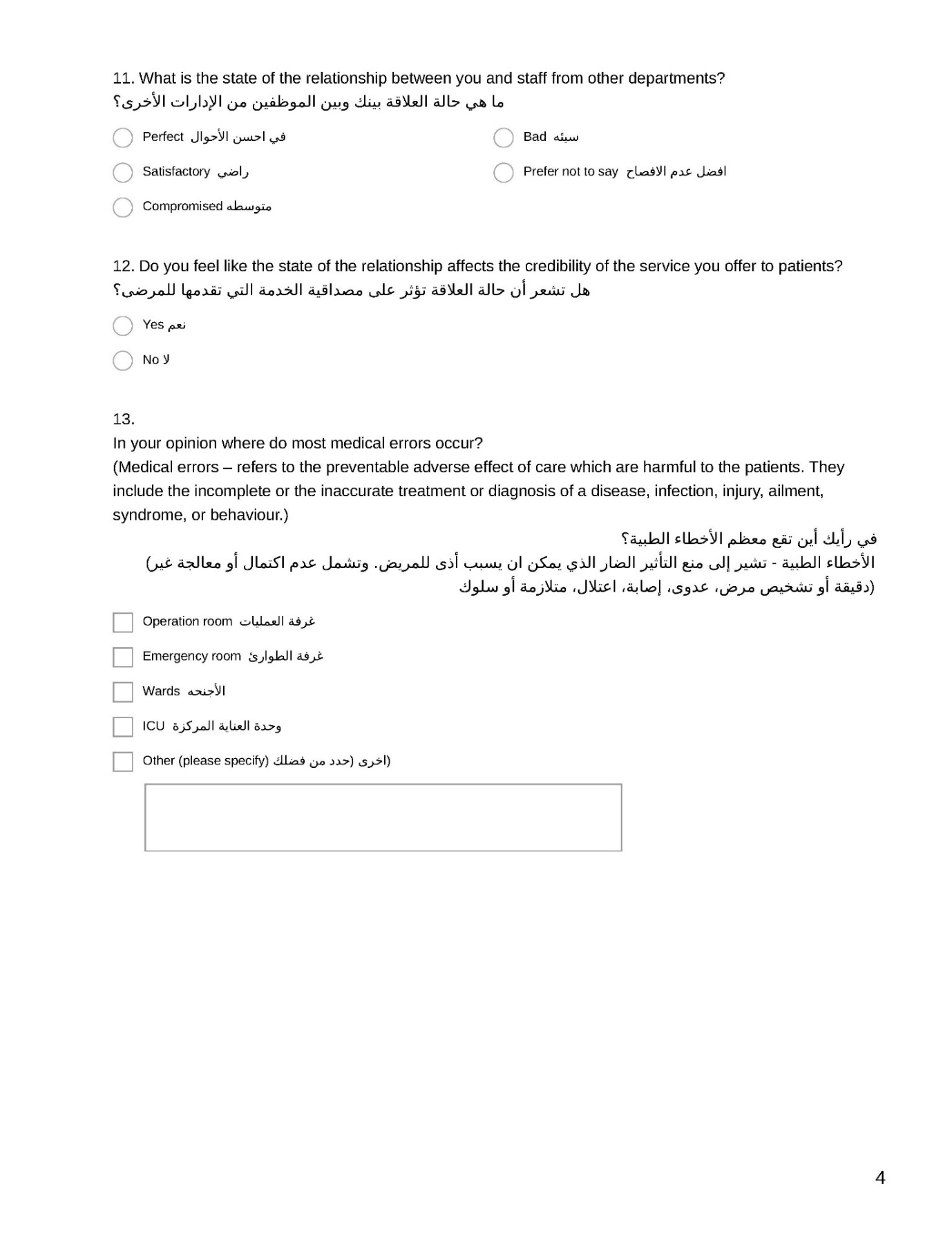


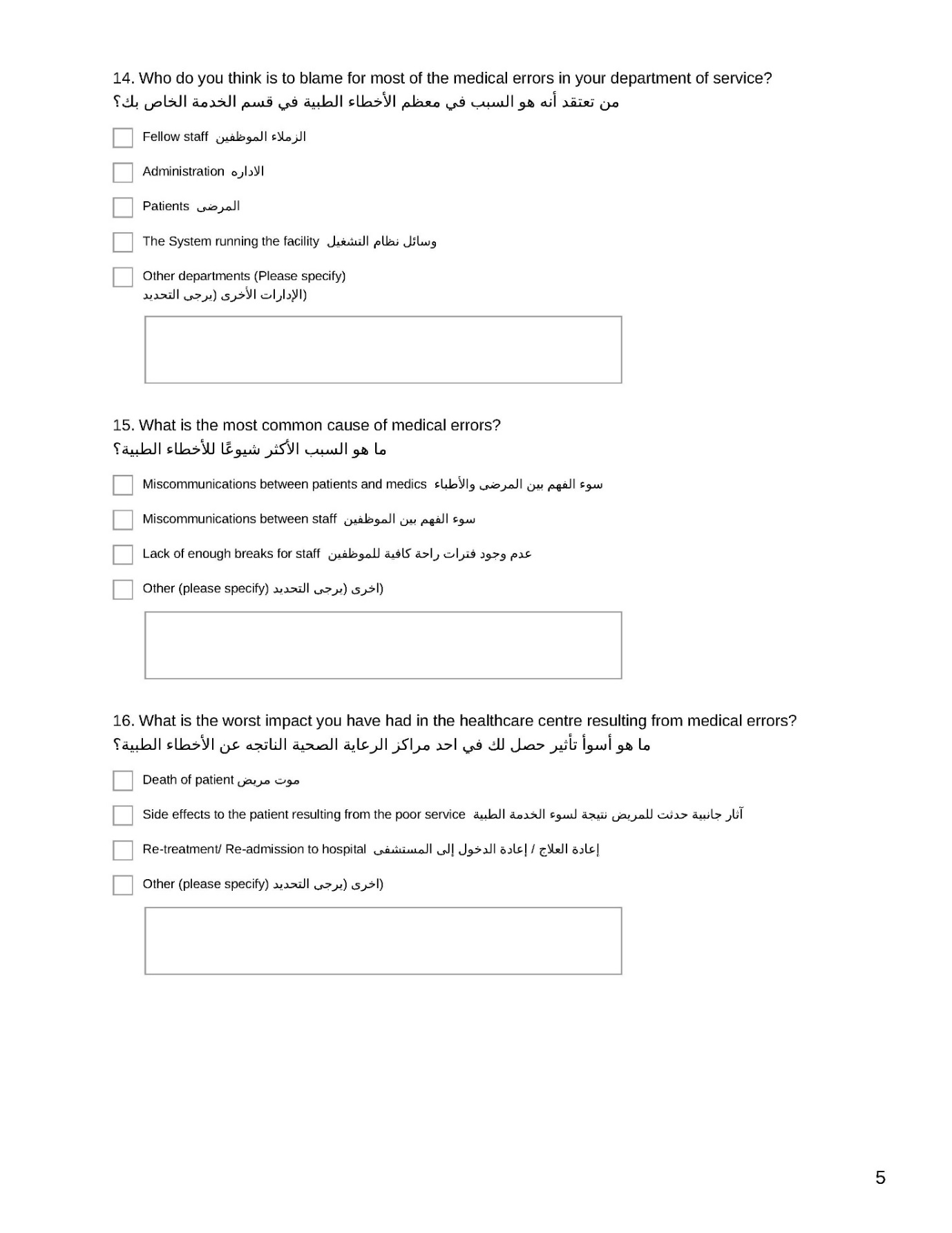


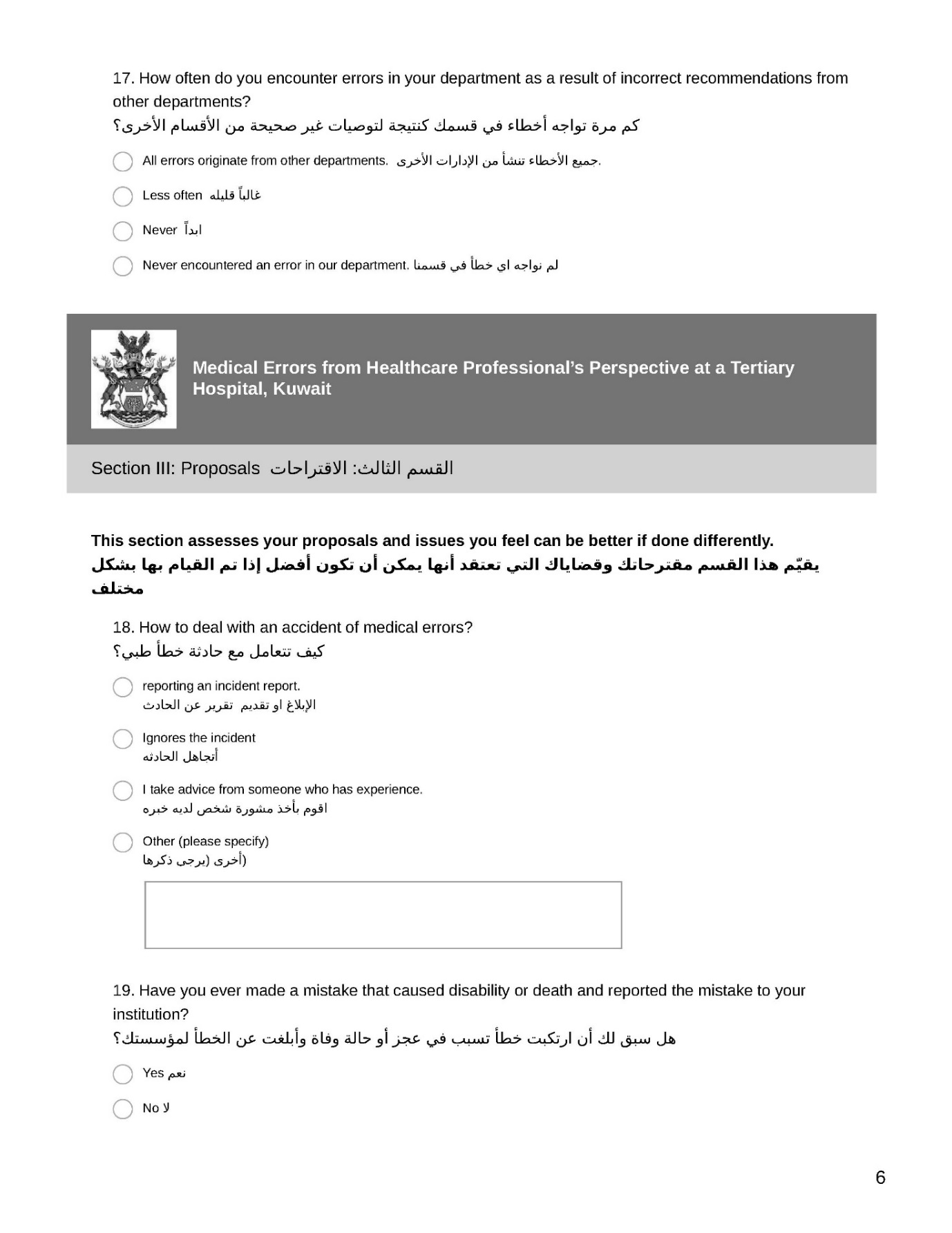


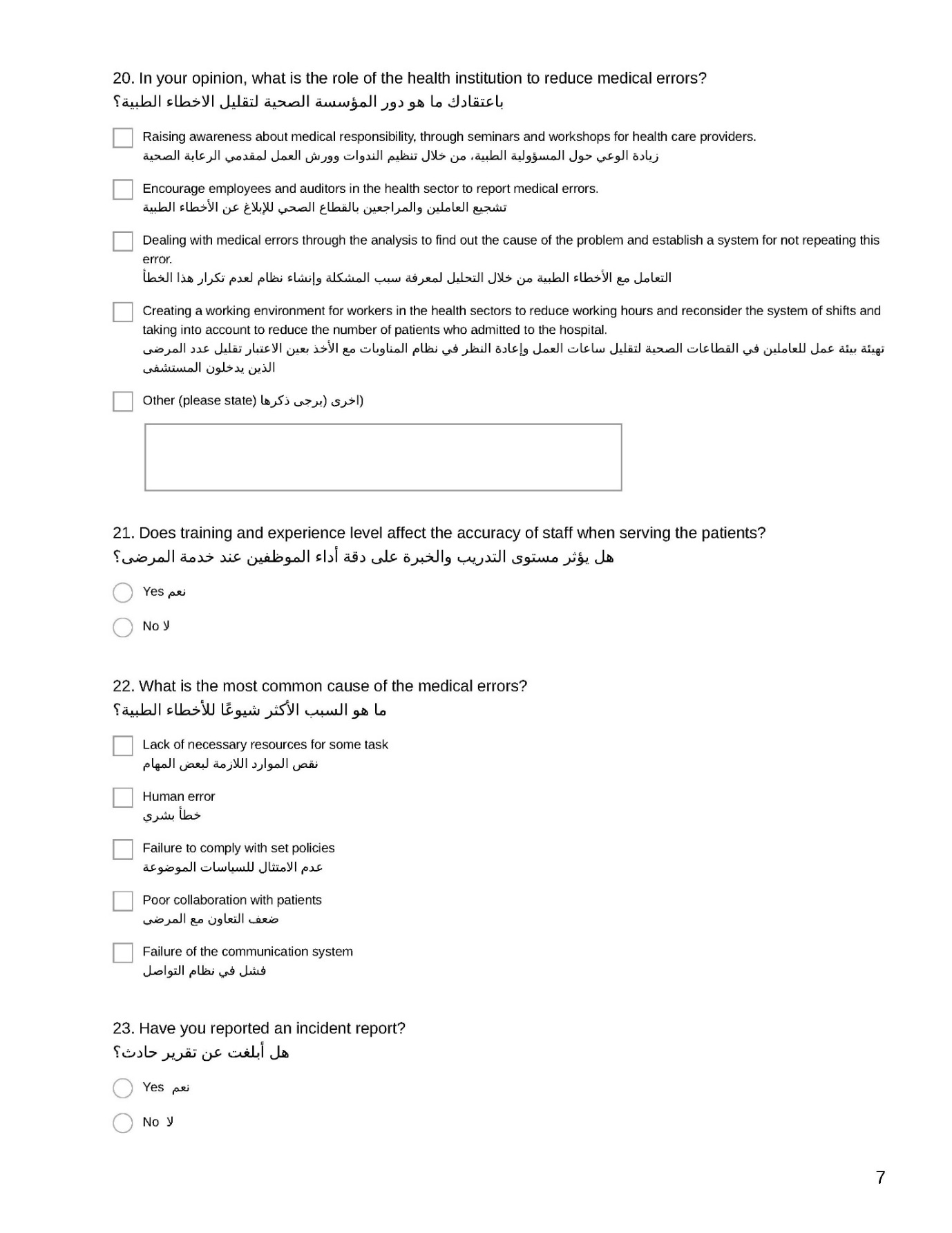


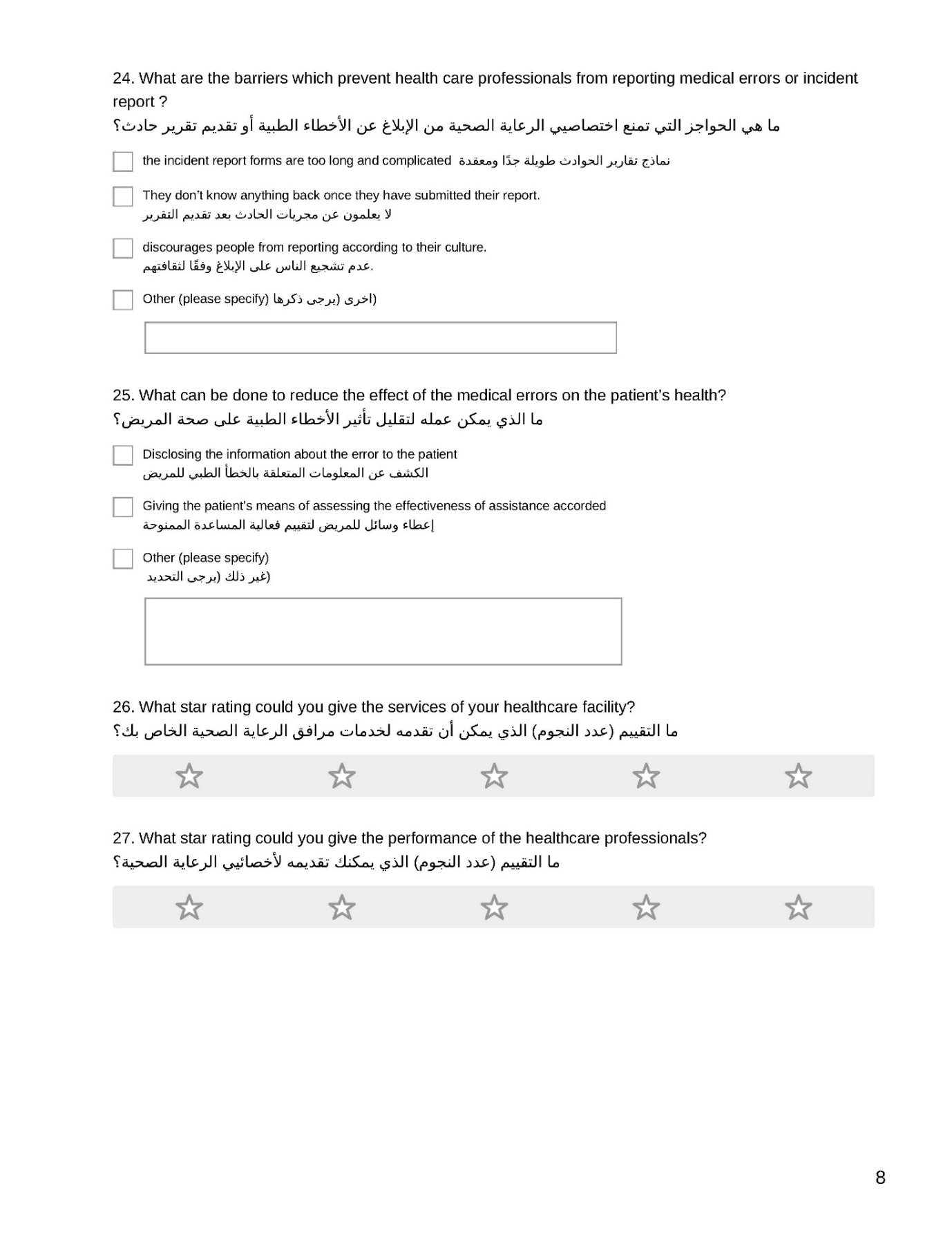

Supplement: S1 Appendix — (DOCX) [file pone.0217023.s001.docx]
